# Complementary Feeding Approach and Maternal Communicative Functions During Mealtimes in 12‐Month‐Old Infants

**DOI:** 10.1111/infa.70060

**Published:** 2025-12-15

**Authors:** Alice Di Prete, Mariarosaria Ciolli, Elisa Iaboni, Noemi Palladino, Giulia Pecora, Melania Paoletti, Valentina Focaroli, Barbara Caravale, Serena Gastaldi, Elsa Addessi, Francesca Bellagamba, Emiddia Longobardi

**Affiliations:** ^1^ Sapienza Università di Roma Rome Italy; ^2^ Università degli Studi di Roma “Tor Vergata” Rome Italy; ^3^ Università degli Studi “Niccolò Cusano” Rome Italy; ^4^ Istituto di Scienze e Tecnologie della Cognizione Rome Italy

## Abstract

The literature on alternative approaches to complementary feeding, especially Baby‐Led Weaning (a complementary feeding approach in which infants participate in family meals and eat finger food independently), has gradually increased in recent years. The present study aims to investigate whether there is a relation between the complementary feeding approach chosen by parents (Baby‐Led Weaning or Parent‐Led Weaning, in which infants are fed puréed food on a spoon) and maternal communicative functions produced during a typical meal. We analyzed the transcripts of mother‐infant interactions occurring during mealtimes of 136 12‐month‐old typically developing infants, by means of a validated coding scheme that allows to identify five different communicative functions (Tutorial, Didactic, Conversational, Control and Asynchronous). The results highlighted a slightly different pattern of communicative style depending on the feeding method adopted by the parents (BLW vs. PLW). In particular, mothers of infants exposed to the PLW approach used the Tutorial function more often than mothers of infants exposed to the BLW method. The two groups did not differ in their use of the other functions. The implications for the promotion of healthy eating behaviors within family interactive contexts are discussed.

## 1 Introduction

In Western countries, infants are usually introduced to solid foods by parents feeding them specifically prepared puréed food on a spoon (Parent‐Led‐Weaning, PLW; Cameron et al. 2013); however, alternative approaches, known as Baby‐Led Weaning (BLW; Rapley and Murkett 2008) or On‐demand complementary feeding (*Alimentazione Complementare a Richiesta*, ACR; Piermarini 2008) have recently emerged. Children exposed to these approaches participate ‐ from the onset of the complementary feeding period (around 6 months of age; World Health Organization 2023)—in family meals and independently eat textured foods proposed by their parents (Brown and Lee 2011) or actively requested by the children themselves (Piermarini 2008). According to the current literature, these alternative feeding methods have positive relations with child development in several domains. The BLW approach shows positive associations with better self‐regulation of food intake (Rowan and Harris 2012), increased participation in family meals (Brown and Lee 2011), and advantages in language (Webber et al. 2021; Farrow et al. 2025; Pecora et al. 2025) and motor development (Addessi et al. 2021; Campeau et al. 2021). Moreover, mothers of 8‐ to 24‐month‐old infants who were more often allowed to eat autonomously (a characterizing feature of BLW) were observed to be more responsive to their fullness cues (Di Prete et al. 2023, 2025). This suggests an increased parental focus on children's behavioral cues and needs during the meal for infants more frequently eating unaided, as recommended by the latest World Health Organization guidelines (World Health Organization 2023).

Mealtime is an important and mandatory context occurring multiple times a day in a child's life: mealtimes involve consistent and predictable social interactions with family members, and can be considered a learning tool to build relations with others (Spegman and Houck 2005). The average infant spends 11.8 h per week eating at home (Hofferth and Sandberg 2001) and eating with family members offers an opportunity for social interactions and modeling which can support linguistic development (Snow and Beals 2006).

A number of studies have shown the effect of environmental factors on communicative style in interactional settings, highlighting the variability of infant‐directed speech during daily family exchanges. Some of these studies have examined verbal exchanges between caregivers (often mothers) and young infants during meals, usually comparing this setting with other contexts, such as playtime, book reading sessions, and other everyday routines (dressing up and bathtime) (Adi‐Bensaid et al. 2022; Han et al. 2021; Tamis‐LeMonda et al. 2019; Majorano et al. 2013; Hoff 2010; Longobardi 1992, 2006; Hampson and Nelson 1993; Hoff‐Ginsberg 1991; Rondal 1980). The results of the above studies are mostly consistent in showing that children use language differently and develop different skills in each setting. Compared with other contexts (especially book reading sessions and playtime), mealtime involves fewer vocal exchanges, and the exposure to language in this context positively predicts children's vocabulary development (Dickinson and Tabors 2001; Weizman and Snow 2001; Beals 1997). Weizman and Snow (2001) recorded and analyzed the conversations of 53 mother‐child dyads in five different contexts (two types of play, two types of book reading, and mealtimes) when children were 5 years old. The mealtime and information‐book conversations generated the greatest amount of maternal talk (800 word‐tokens). Mealtime conversations were the longest (averaging 20 min, including periods of silence) and generated the highest average number of sophisticated word‐tokens (low‐frequency vocabulary) with an average of 11 per conversation. However, mealtime conversations generated less maternal language input (40 word‐tokens per minute) than the other contexts (for instance, information‐book conversations generated 112 word‐tokens per minute).

Another line of research investigated the pragmatic features of maternal interactional style, showing that mother's speech can vary in different contexts and can be used for several purposes, for example to elicit conversations (McDonald and Pien 1982), to direct infants' attention and action (Flynn and Masur 2007; Landry et al. 2006), to provide new information (Bornstein et al. 1992). Flynn and Masur (2007) found that mothers used more descriptions of objects and actions that followed the child's attention during play sessions than during the bath routine in the first 2 years of age. Longobardi et al. (2022) compared maternal communication style by analyzing the use of five communicative functions (Tutorial, Didactic, Conversational, Control and Asynchronous) in two contexts (free play and meal) in 16‐month‐olds. These five dimensions let to distinguish maternal behavior in different pragmatic purposes: Tutorial function (comments that support the child's attempts to communicate, extend, and reformulate child's verbal or non‐verbal expressions), Didactic function (comments that provide the child with new information about objects and events), Conversational function (comments that promote and maintain the communicative exchange with the child), Control function (comments that direct or modify the ongoing action and/or attention of the child), and Asynchronous function (comments that ignore or are clearly incongruent with the child's focus of action and attention). The results showed significant contextual differences for three of the five communicative functions. In particular, the Control function was more frequent during free play than during mealtimes, indicating a greater attitude to direct the activities in which the child was more involved. Moreover, the Asynchronous function was produced significantly more often in the context of free play than during mealtimes. Although the authors suggested caution in interpreting this result, since the frequency of the Asynchronous function was extremely low (especially during mealtimes), it seems that mothers had significantly more difficulty following their child's focus of action during play activities. On the contrary, the Conversational function was used more frequently during mealtimes than during free play sessions, perhaps because children tend to talk less while eating, and therefore mothers talked more to promote conversation. Finally, although the Didactic and Tutorial functions were more frequent during play than during mealtimes, these differences were not significant.

Another important dimension of meal routines, that is specific to feeding interaction, is the level of control that parents exercise on what and how much the infant is eating. Since the overall goal of the meal is nurturing the infant, one important aspect to look at is the level of autonomy in feeding that the infant is allowed by parents. In this respect, from the literature we know that control dominance between mother and child during mealtimes can vary depending on parenting styles (Baumrind 1967), maternal rules (Houck and LeCuyer‐Maus 2002), child compliance (Crockenberg and Littman 1990), self‐regulation (Houck and LeCuyer‐Maus 2004), and childhood eating patterns (Valtolin and Ragazzoni 1995). It is in the early years of life that young children learn to balance autonomy and obedience, and mealtimes are a central context for this kind of negotiation of control and social behavior learning (Spegman and Houck 2005).

However, to our knowledge, no study has yet explored whether maternal communicative functions are associated with the kind of complementary feeding approach chosen by caregivers, which can vary along a continuum from a strictly parent‐led weaning approach, up to a strictly baby‐led weaning approach, depending on the proportion of self‐feeding episodes that the infant is allowed during the meal. To this end, the specific aim of the present study was to investigate the use of different communicative functions, such as Tutorial, Didactic, Conversational, Control and Asynchronous, according to the complementary feeding approach adopted by mothers of 12‐month‐old infants. Based on the literature reviewed, the choice to analyze the five functions was due to the possibility of detecting the widest range of variability in maternal communicative behaviors during interactions with the infant. Furthermore, given the greater sharing of meals within families adopting the BLW approach, the participation of other family members during the meal was also taken into consideration in our investigation (Rapley and Murkett 2008; Brown and Lee 2011).

More specifically, as a first exploratory analysis, our research questions were as follows.

- Do mothers in the two groups of complementary feeding approach (BLW and PLW) differ in the use of communicative functions?
- Does the use of communicative functions vary in relation to the structural characteristics of the meal situation, that is, in relation to the participation of other family members during the meal?

## 2 Methods

### 2.1 Participants

Participants were 182 Italian mothers with their 12‐month‐old infants. Data was collected in the context of a broader longitudinal study investigating the relation between complementary feeding approach and infant psychomotor and linguistic development in the first 2 years of life. Data on linguistic development (maternal child‐directed speech and infant gestural and verbal communication) have been previously reported by Pecora et al. (2025). For the present study, we focused only on children who were more strictly exposed to Baby‐Led‐Weaning (52.2% of the participants, showing a percentage of self‐feeding > 70%) and on children who were more strictly exposed to Parent‐Led Weaning (47.8% of the participants, showing a percentage of self‐feeding < 40%), thus excluding those children exposed to a mixed complementary feeding approach. This reduced the number of participants from 182 to 136. The sample size for the present study was determined as part of a larger longitudinal project (Spoon Project; Bellagamba et al. 2020).

Both parents provided written parental consent for taking part in the study and to be video recorded. All procedures were approved by the Ethics board of the Department of Dynamic and Clinical Psychology and Health Studies of Sapienza University of Rome (Prot. N. 0000315, April 14, 2020, and n. 0001209, December 15, 2020) and by the Research Ethics and Integrity Committee of the National Research Council of Italy (Prot. N. 00721482019, October 18, 2019, and n. 0028810, April 23, 2021).

### 2.2 Procedure

We analyzed the transcripts of mother‐infant conversations occurring during a meal of 136 12‐month‐old typically developing infants (45.6% females). As reported above, a typical meal for each participant was previously recorded through Skype or JitsiMeet and analyzed by using the ELAN software (Max Planck Institute for Psycholinguistics, the Language Archive 2024) by Pecora et al. (2025). All intelligible speech addressed by mothers to the infants was verbatim transcribed and later coded for communicative functions (see paragraph 2.3.2 below).

### 2.3 Measures

#### 2.3.1 Sociodemographic Variables and Feeding Measures

In Pecora et al. (2025), when infants were 12 months old, mothers completed an online survey to provide information on sociodemographic variables, breastfeeding habits, and the complementary feeding approach employed. In Pecora et al. (2025), from the recordings of mealtimes, we also evaluated the proportion of self‐feeding (i.e., the number of episodes in which the infant ate autonomously divided by the total number of feeding episodes). The subjects were 136 non‐bilingual infants with typical development. Most of the mothers (76.47%) were employed and had a university education (87.5%). Only two mothers were not born in Italy but had lived there for more than 5 years. Italian was always the main language spoken in the house. Data about household income was inconsistent and therefore not reported. Overall, however, the socioeconomic level was medium‐high. All data were collected in Italy, and all participants were Italian.

#### 2.3.2 Maternal Communicative Functions

Maternal child‐directed speech was analyzed considering five communicative functions (Tutorial, Didactic, Conversational, Control and Asynchronous) that reflect different pragmatic properties of maternal interactive styles, by using a coding scheme including 21 categories developed and employed in previous studies (Camaioni et al. 1998; Longobardi et al. 2018, 2022; Majorano et al. 2013). These behavioral categories cover a continuum that ranges from maternal utterances that are highly synchronized and well‐adapted to the child's focus of attention and ongoing actions to maternal utterances that are poorly or not at all attuned to the child's line of attention/action (Longobardi 1992). Categories 1–7 define the Tutorial function, which involves highly synchronized and well‐adapted maternal interventions on the child's attention or action, with the aim of providing support to the activity and taking it forward to progressively more advanced levels (repetition, expansion/extension, reformulation, paraphrase, reference to a shared experience, reference to the roles of a social game, compliment/encouragement). Categories 8–12 define the Didactic function, which involves the transmission of information to the child or verifies the knowledge or skills he/she already has (description/demonstration, closed question, request for repetition, labeling, correction). Categories 13–16 define the Conversational function which involves the promotion and continuation of communicative exchanges with the child (conversational prompts, open questions, empathetic comment, self‐response). Categories 17–18 define the Control function, which involves redirecting the child's attention or modifying the action the child is occupied with (action directive, attention directive). Categories 19–21 define the Asynchronous function, which involves interventions that are not synchronized with the child's attention or action, to the point of ignoring or distorting it (overlapping behavior, change of topic, ignoring child's initiative). These categories are mutually exclusive; thus, each maternal utterance could be assigned to one and only one category. Since the meals varied in duration (mean length = 20.3, SD = 10.8), according to the habits of each family, we considered the frequency per minute of each of the five communicative functions reported above.

### 2.4 Inter‐Rater Reliability

ADP was trained in the use of the coding scheme for communicative functions by EL during several in‐person meetings, and they reviewed together a portion of the transcripts. Throughout the coding process, EL was available to clarify any doubts. EA and FB (experts in the analysis of parent‐child interaction) attended the meetings and intervened to resolve the few disagreements.

### 2.5 Statistical Analyses

First, we assessed whether the duration of the meal and the number of mother's communicative acts toward the infant by minute differed according to the complementary feeding approach (BLW, PLW) by means of Wilcoxon signed‐ranks tests. We then analyzed whether child sex, presence of the mother‐child dyad during the meal (as compared to meals in which also other people were present), complementary feeding approach, duration of exclusive breast‐feeding (months), and total number of communicative functions by minute were related to the communicative functions by means of linear regression models. Statistical analysis was performed using Stata 14 software (StataCorp LP 2020, College Station, TX). The significance level was set at *p* < 0.05.

## 3 Results

When considering the feeding method, 52.2% of mothers adopted Parent‐Led‐weaning (i.e., their children self‐fed in < 40% of the feeding episodes), and 47.8% Baby‐Led‐weaning (i.e., their children self‐fed in > 70% of the feeding episodes). In 45.6% of meals only the mother‐child dyad was present (See Table 1 for more details).

**TABLE 1:** Demographic information (*N* = 136).

Variable	Values
Infant age (months, mean ± SD)	12.4 ± 0.47
Infant sex (*n* and %)	
Females	62 (45.6%)
Males	74 (54.4%)
Siblings (*n* and %)	
Yes	51 (37.5%)
No	85 (62.5%)
Maternal age (years, mean ± SD)	35.9 ± 3.82
Mother employment (*n* and %)	
Yes	104 (76.47%)
No	28 (20.59%)
Missing	4 (2.94%)
Maternal education (*n* and %)	
Batchelor degree or higher	119 (87.5%)
Not a university degree	14 (10.3%)
Missing	3 (2.2%)
Age at the onset of complementary feeding (months, mean ± SD)	5.61 ± 0.74
Complementary feeding method (*n* and %)	
PLW	71 (52.2%)
BLW	65 (47.8%)
Children attending daycare (*n* and %)	
Yes	51 (37.50%)
No	81 (59.56%)
Missing	4 (2.94%)
Proportion of self‐feeding (mean ± SD)	0.50 ± 0.42

Abbreviations: BLW, baby‐led weaning; PLW, parent‐led weaning.

In the whole sample, communicative functions were distributed as follows: 32.68% Tutorial function, 29.27% Control function, 25.66% Conversational function, 12.26% Didactic function, and 0.13% Asynchronous function. The means and standard deviations for each communicative function (presented as frequency by minute) are shown in Table 2.

**TABLE 2:** Means and standard deviations for each communicative function (frequency by minute) (*N* = 136).

Variables	Total sample	BLW	PLW
Tutorial function	3.00 ± 2.07	2.26 ± 1.84	3.69 ± 2.05
Didactic function	1.09 ± 0.83	0.99 ± 0.78	1.19 ± 0.87
Conversational function	2.50 ± 1.94	2.21 ± 1.82	2.71 ± 2.03
Control function	2.50 ± 1.6	2.31 ± 1.55	2.65 ± 1.62
Asynchronous function	0.01 ± 5.4	0.01 ± 0.04	0.02 ± 0.09

The BLW meals were significantly longer than PLW meals (*z* = −5.46; *p* < 0.001), although PLW mothers produced more sentences directed towards their children (*z* = 2.78; *p* = 0.005). Table 2 shows the frequency of maternal communicative functions by minute according to the complementary feeding method. The most frequent function by minute in the BLW group was the Control function (Mean = 2.31; SD = 1.55), while the most frequent function by minute in the PLW group was the Tutorial function (Mean = 3.69; SD = 2.05). In both groups the frequency by minute of the Asynchronous function was very low (BLW: Mean = 0.01, SD = 0.04; PLW: Mean = 0.02, SD = 0.09).

When we considered the influence of other factors on the maternal communicative functions, we found that only for the Tutorial function there was a significant effect of the complementary feeding approach, in that this function was more frequent in the PLW group than in the BLW group (*z* = −3.55; *p* = 0.001); additionally, the Tutorial function was more frequent when only the mother and the child were present than when other people joined the meal (*t* = −2.25; *p* = 0.026). Table 3 reports the full models for all variables.

**TABLE 3:** Linear regression analyses with communicative functions as dependent variables.

Variable	Coeff.	Robust. SE	*t*	*p*	95% conf. Interval	
Tutorial function						
Gender	−0.16	0.19	−0.82	0.411	−0.55	0.22
Dyad presence	−0.47	0.21	−2.25	**0.026**	−0.88	−0.06
Complementary feeding method	−0.70	0.19	0.3.55	0.001	−1.07	−0.30
Exclusive breastfeeding	−0.0004	0.04	−0.01	0.993	−0.09	0.09
Number of total functions by minute	0.33	0.02	13.53	**0.000**	0.28	0.38
Intercept	0.61	0.31	1.99	0.049	0.002	1.22
*R* ^2^	0.76***					
Didactic function						
Gender	−0.02	0.10	−0.17	0.452	−0.21	0.18
Dyad presence	0.08	0.11	0.75	0.869	−0.13	0.29
Complementary feeding method	0.10	0.11	1.02	0.311	−0.10	0.32
Exclusive breastfeeding	0.005	0.02	0.26	0.798	−0.04	0.05
Number of total functions by minute	0.12	0.01	10.21	**0.000**	0.09	0.14
Intercept	−0.11	0.13	−0.88	0.379	−0.37	0.14
*R* ^2^	0.63***					
Conversational function						
Gender	0.17	0.15	1.08	0.283	−0.14	0.47
Dyad presence	0.32	0.17	0.185	0.067	−0.02	0.67
Complementary feeding method	0.28	0.18	1.59	0.115	−0.07	0.63
Exclusive breastfeeding	0.03	0.03	0.86	0.390	−0.04	0.09
Number of total functions by minute	0.31	0.02	15.38	**0.000**	0.27	0.35
Intercept	−0.83	0.22	−3.77	0000	−1.28	−0.40
*R* ^2^	0.79***					
Control function						
Gender	0.002	0.17	0.02	0.987	−0.34	0.34
Dyad presence	0.05	0.20	0.28	0.783	−0.34	0.45
Complementary feeding method	0.30	0.21	1.43	0.156	−0.12	0.72
Exclusive breastfeeding	−0.03	0.04	−0.79	0.433	−0.12	0.05
Number of total functions by minute	0.23	0.02	9.77	**0.000**	0.18	0.28
Intercept	0.34	0.25	1.35	0.180	−0.16	0.85
*R* ^2^	0.63***					
Asynchronous function						
Gender	0.007	0.009	0.80	0.426	−0.01	0.02
Dyad presence	0.008	0.009	0.83	0.407	−0.01	0.03
Complementary feeding method	−0.001	0.014	−0.09	0.930	−0.03	0.03
Exclusive breastfeeding	0.0002	0.004	0.06	0.956	−0.007	0.008
Number of total functions by minute	0.001	0.001	1.49	0.139	−0.0005	0.003
Intercept	−0.007	0.018	−0.39	0.696	−0.04	0.03
*R* ^2^	0.03					

*Note:* Values in bold are significant, ***p < 0.001.

## 4 Discussion

This study examined mothers' use of five communicative functions (Tutorial, Didactic, Conversational, Control, and Asynchronous) during mealtime interactions with their 12‐month‐old infants, taking into account the complementary feeding approach used (Baby‐Led Weaning vs. Parent‐Led Weaning). We also examined whether the use of maternal communicative functions differed when only the mother and the infant were present as compared to when other people joined the meal.

In comparison with a previous study that investigated mothers' communicative functions during mealtimes and free play (Longobardi et al. 2022), considering only mealtime observations the Tutorial function was observed more frequently (32.68% vs. 13.99%), while the Conversational function was observed less frequently (25.66% vs. 44.81%). This could be due to several factors such as the lower sample size of the previous study (which involved only 25 mothers), the different age of the children (who were 16 months old) and, most notably, the fact that in the previous study communicative functions were investigated independently of the complementary feeding approach. Instead, the present study specifically analyzed, for the first time, whether maternal communicative functions differed between groups using either BLW or PLW complementary feeding approaches.

The meals of infants exposed to the BLW approach were longer than those of infant exposed to PLW, likely because BLW children need more time to self‐feed (Rapley et al. 2015); however, despite this, PLW mothers were found to be producing more child‐directed sentences than BLW mothers. When controlling for the number of mother's communicative acts toward the infant, the frequency of virtually all communicative functions was rather similar between the BLW and PLW complementary feeding approaches, showing that both approaches offer a responsive communicative style to the infants during mealtimes. Only for the Tutorial function, the two complementary feeding groups differed significantly. Specifically, mothers who chose the PLW approach used the Tutorial function more frequently than those who followed the BLW approach. While BLW children, especially when they are 12 months old, usually eat independently, PLW infants are often still spoon‐fed and may still need some encouragement and support during meals. This hypothesis is supported by the observation that the Tutorial function was more frequent in dyadic meals, which are usually more frequent in PLW.

Previous work has documented that dyadic vs triadic interactive situations differ for important pragmatic factors. When the interactive situation is triadic, as in the case of twins, children received less speech directed specifically to them, participated in fewer and shorter episodes of joint attentional focus, and had fewer and shorter conversations with their mothers (Longobardi et al. 2001; Tomasello et al. 1986). Indeed, when involved in a family meal, mother must divide her time and attention between more than one participant simultaneously and may offer less scaffolding and support to the one‐year‐old target child.

The observation that the Control function was the most frequent one in the BLW group was a surprising result because of the higher degree of independence that characterizes the BLW approach. However, two possible explanations may be proposed. First, while mothers who chose the PLW approach exercise a certain degree of physical control on their infants during feeding activities (by deciding what, how often and how much food they offer on a spoon; Brown and Lee 2011) without needing to verbalize it, mothers who chose the BLW approach allow the child to be physically autonomous while eating and may need to supplement their parental role with more verbal directives, thus using the Control function more frequently. A second possible explanation is that PLW children do not have to coordinate as many actions while eating as self‐feeding children but have just to open and close their mouth in response to the food offered by the caregiver (Sachs 2011). In contrast, children that are learning to self‐feed need to coordinate more movements and behaviors and consequently may make more failed attempts to eat. Therefore, their mothers may need to use more verbal control to support and guide their child's uncertain feeding attempts and maintain their attention focused on the fulfillment of the goal of eating.

Another noteworthy result concerns the very low use of the Asynchronous function by mothers belonging to both feeding groups. This highlights how there were no significant dysfunctional interactive episodes related to the complementary feeding approach, thus preserving a good degree of maternal responsiveness to the infant. Furthermore, this result is in agreement with previous studies that have found a very low frequency of maternal Asynchronous function in both meal and play contexts (Longobardi 1992; Longobardi et al. 2022).

The opportunity to assess mother‐child interaction directly from recorded transcripts (rather than by questionnaires or interviews) is a major strength of this study. However, our study also has some potential limitations. During most meals, fathers were not present, since we asked parents to record a typical meal, without mentioning if one or more caregivers should be present; however, the father's role could be worth further exploration in future studies, by asking parents to record a meal when the entire family is present.

In conclusion, the frequency of the different communicative functions was rather similar between the BLW and PLW complementary feeding approaches, apart from a significantly higher frequency of the Tutorial function in the PLW group, possibly explained by the higher frequency of dyadic meals in this group. In future studies, it could be interesting to analyze if the difference in the expression of maternal communicative functions between BLW and PLW groups is maintained when children grow older and are involved in other activities, such as play.

## Author Contributions

**Alice Di Prete:** data curation, formal analysis, investigation, methodology, writing – original draft. **Mariarosaria Ciolli:** investigation. **Elisa Iaboni:** investigation. **Noemi Palladino:** investigation. **Giulia Pecora:** investigation. **Melania Paoletti:** investigation. **Valentina Focaroli:** investigation. **Barbara Caravale:** conceptualization. **Serena Gastaldi:** data curation. **Elsa Addessi:** conceptualization, data curation, formal analysis, funding acquisition, methodology, project administration, supervision, writing – original draft, writing – review and editing. **Francesca Bellagamba:** conceptualization, funding acquisition, methodology, project administration, supervision, writing – original draft, writing – review and editing. **Emiddia Longobardi:** conceptualization, methodology, supervision, writing – review and editing.

## Funding

Funded by the European Union ‐ Next Generation EU, Mission 4 Component 1 CUP B53D23014810006 and B53D23014820006 (PRIN 2022 Grant No. 2022S8PEY7) and by the Italian Ministry of Education, University and Research, Progetti di Rilevante Interesse Nazionale (PRIN 2017), Grant No. 2017WH8B84. Alice Di Prete was funded by a PHD fellowship from the European Union ‐ Next Generation EU, Mission 4 Component 1 CUP B53C23002130006.

## Ethics Statement

The study was conducted in accordance with the Declaration of Helsinki, and approved by the Ethics board of the Department of Dynamic and Clinical Psychology and Health Studies of Sapienza University of Rome (Prot. N. 0000315, April 14, 2020, and n. 0001209, December 15, 2020) and by the Research Ethics and Integrity Committee of the National Research Council of Italy (Prot. N. 00721482019, October 18, 2019, and n. 0028810, April 23, 2021).

## Conflicts of Interest

The authors declare no conflicts of interest.

## Data Availability

Data will be made available on request.
